# Reports on the Hospitals of the United Kingdom

**Published:** 1910-05-21

**Authors:** Henry Burdett


### REPORTS

ON THE

### Hospitals of the United Kingdom.

By SIR HENRY BURDETT, K.C.B., K.C.V.O.

### XLVII.—MANCHESTER CHILDREN'S HOSPITAL, PENDLEBURY.

This hospital was founded in 1829. The present hospital was built upwards of thirty years ago; the Victoria Ward was opened in 1902 and a new out-patient department constructed in 1907. The hospital contains at the present time 168 beds, with an average occupancy of 128. Connected with the institution is a convalescent home at St. Anne's-on-Sea, and a private nursing institution. Other special features include a horse ambulance, a new operation-theatre, and five spacious and excellent verandahs, all of which have been recently erected.

### Its General Plan.

The general plan of this hospital is attractive and good. In the front, facing the road, stands the administration block. Entering this by the centre, we at once see a magnificent and well-proportioned central corridor, which runs the whole depth of the site. On either side of this corridor are three pavilions. Between the first and second pair is placed the housekeeper's department, with ample accommodation for linen, and the dispensary. The Nurses' Home stands between the middle and end pavilions. This Home is divided by the central corridor into two parts. At the extreme end of the site is the laundry and general washhouse, whilst the mortuary and stables occupy a position at the right-hand top corner, well away from the hospital proper.

### Interior Arrangements and Management.

Formerly the end block was set apart for the treatment of infectious cases. This arrangement gave infinite trouble for years, for the hospital was seldom free from outbreaks of infectious disease. Some years ago the fever block was closed, and the whole of the patients were removed to the Monsall Fever Hospital, where all cases of an infectious character occurring in Manchester are now dealt with. What was once a fever block has been adapted for the reception of ordinary cases, and forms one of the pavilions of the Children's Hospital proper.

No one can visit this hospital without being struck by the beauty of many of its interior arrangements; while its grounds and gardens form a special feature, and are probably unequalled by those of any similar institution in the world. The administration of the Manchester Children's Hospital has always been good—a statement based upon personal knowledge which has extended over a period exceeding thirty years. Everything was in the most perfect order, and we could not find a single point which called for criticism or a single instance where the regulations were not strictly enforced. Every part of the hospital was spotlessly clean and smartly kept; all the sisters were keenly interested in their work and exceptionally efficient; the nurses were all most attentive and kind, so that the children regard them with evident affection and confidence; and the whole place possesses a home-like atmosphere, for everyone appears to do their best to promote harmony and comfort, and to consider everybody's claims before their own. We think, as a matter of justice, that we are entitled to say, after a most careful inspection, that the Manchester Children's Hospital is about the best institution of its kind in the world.

One sure test of the relative efficiency of the Children's Hospital is to stand in the centre of the pavilions or buildings and to notice if any, or how many, of the children are crying. A child never cries unless it has cause to do so, and it is the perfection of management and nursing so to understand the children that no child is permitted to remain in an avoidable state of discomfort, a fact which is too little recognised by nurses and others who have charge of children in some children's hospitals. We know hospitals in various places where the children are always crying, and a close inspection has convinced us that the fault lay not with the patients but with those whose duty it is to minister to their necessities and to see to their comfort and well-being. Children, so far as our observation goes, seldom or never cry at Pendlebury, but the poor youngsters whom fate has consigned to the Hope Hospital can frequently be heard from the roadway remonstrating through tears at the hard lot which has befallen them, owing mainly to the failure of the Guardians to provide, for their care and comfort, an adequate staff of nurses and a sufficient equipment of beds, bedding, clothing, and other requisites. We commend these practical considerations to the earnest attention of every member of a committee of a children's hospital throughout the country.

### The Resident Staff.

Pendlebury has more than a local reputation, for its splendid efficiency has made it known throughout Great Britain and in the United States of America. This high reputation has attracted a number of able men and women, who form the present committee and resident staff. It is a pleasure to meet any one of those responsible for the management, for they are all imbued with the same excellent spirit, are keenly interested in their work, and there is not one of them who does not put the interests of the children and the well-being of the institution before all other considerations. We have already spoken of the sisters, and many an old Pendlebury sister now occupies an important position in one of the larger voluntary hospitals. Miss Cameron, the lady superintendent, who was trained at the Western Infirmary, Glasgow, and has held important posts at the Bradford Infirmary and at the Children's Hospital, Bradford, is a very able administrator, full of energy and knowledge, and well qualified to discharge efficiently the duties of one of the highest positions in the nursing world. The Secretary (Mr. H. J. Eason) is a young, energetic, and successful man, who puts life into everything he touches, and has proved a genuine success in his present office.

**The Royal Albert Edward Infirmary, Wigan,** will b e reported on next week.

